# Accuracy of the serological ELISA test compared with the polymerase chain reaction for the diagnosis of cytomegalovirus infection in pregnancy

**DOI:** 10.1590/S1516-31802003000300002

**Published:** 2003-05-01

**Authors:** Silvana Varella Parmigiani, Ricardo Barini, Sandra Cecília Botelho Costa, Eliana Amaral, José Carlos Gama da Silva, João Luiz de Carvalho Pinto e Silva

**Keywords:** Virus, Pregnancy, Serology, Polymerase chain reaction, Vírus, Gestação, Sorologia, Reação de polimerase em cadeia

## Abstract

**CONTEXT:**

The most frequently used methods for detecting antibodies are the indirect immunofluorescence test and the enzymatic immunoassay (ELISA). The polymerase chain reaction is a molecular biology technique in which the production of large amounts of specific DNA fragments is induced from very low concentrations of complex substrates aloowing the detection of very low amounts of viral particles.

**OBJECTIVE:**

To assess the accuracy of serological/ELISA tests in comparison with the polymerase chain reaction in maternal blood to diagnose cytomegalovirus infection.

**DESIGN:**

A descriptive study was performed.

**SETTING:**

High-risk outpatient clinic of Campinas University (Unicamp).

**PARTICIPANTS:**

We selected 243 pregnant women. All of them had been indicated for blood sampling because of suspicions of cytomegalovirus infection and also because of other infections.

**MAIN MEASUREMENTS:**

The group was tested for cytomegalovirus. Serological tests were run and compared to the polymerase chain reaction, which was considered to be the gold standard. Status analyses were done using Fisher's exact test, via the SAS software.

**RESULTS:**

The previous cytomegalovirus infection rate was 94.6%. The main reasons for inclusion in the study were fetal nervous system malformation (25.5%), maternal toxoplasmosis (25.5%) and Rh isoimmunization (14.8%). Only two women were included because of positive serological immunoglobulin M test for cytomegalovirus. The sensitivity and specificity of the serological tests were 94% and 6% for immunoglobulin G.

**CONCLUSION:**

Serological tests had lower sensitivity in comparison with the polymerase chain reaction test when diagnosing cytomegalovirus infection. The consequences of positive polymerase chain reaction and negative immunoglobulin M in women remain unknown.

## RESUMO

**CONTEXTO:**

Os métodos mais freqüentemente utilizados na detecção de anticorpos são a imunofluorescência indireta e o ensaio imunoenzimático (Elisa). A reação de polimerase em cadeia é uma técnica de biologia molecular em que a produção de grande quantidade de fragmentos específicos de DNA é induzida a partir de baixas concentrações de substratos, permitindo a detecção de pequenas quantidades de partículas virais.

**OBJETIVO:**

O objetivo deste trabalho foi estudar a acurácia do teste sorológico Elisa (*enzyme linked immunosorbent assay*) no diagnóstico de infecção materna pelo citomegalovírus avaliada pela reação de polimerase em cadeia por meio de amostragem de sangue materno.

**TIPO DO ESTUDO:**

Descritivo.

**LOCAL:**

Ambulatório de Pré-Natal Especializado do Centro de Assistência Integral à Saúde da Mulher (PNE/CAISM) da Universidade Estadual de Campinas (UNICAMP).

**PARTICIPANTES:**

243 gestantes atendidas no serviço. As gestantes que constituíam a amos-tra foram aquelas que tinham indicação de análise sangüínea.

**METODOLOGIA E ANÁLISE ESTATÍSTICA:**

As pacientes foram submetidas à coleta de sangue venoso periférico para pesquisa de citomegalovírus utilizando-se comparativa-mente o teste sorológico em relação à reação de polimerase em cadeia. Neste estudo, o padrão-ouro utilizado foi a reação de polimerase em cadeia em amostras de sangue. A análise estatística foi feita pelo teste Exato de Fisher usando o programa SAS.

**RESULTADOS:**

As principais indicações para a análise foram diagnóstico de malformação do sistema nervoso central (25,5%), toxoplasmose materna (25,5%) e isoimunização pelo fator Rh (14,8%). A freqüência de infecção pregressa pelo citomegalovírus foi de 94,6% na população estudada. Duas das pacientes apresentavam suspeita de infecção ativa pelo citomegalovírus por positividade para imunoglobulina M. Comparando os testes diagnósticos em sangue materno, encontramos uma sensibilidade de 94% e especificidade de 6% da imunoglobulina G materna.

**CONCLUSÃO:**

Os testes sorológicos mostraram baixa acurácia diagnóstica em relação à reação de polimerase em cadeia na identificação de gestantes com infecção ativa pelo citomegalovírus na população estudada. As conseqüências da positividade na reação de polimerase em cadeia em imunoglobulina M negativa em gestantes não estão esclarecidas.

## INTRODUCTION

Cytomegalovirus, a DNA virus belonging to the herpes family, infects people from different age groups, races and socioeconomic conditions .^1^ Its prevalence rate ranges from 40% to 60% in many countries where the population has good socioeconomic conditions.^2^ Primary cytomegalovirus infection occurs in 0.7% to 4.1% of pregnancies and the transmission rate varies between 24% to 75%, averaging 40%.^3-8^ Fetal transmission occurs in 0.5% to 1% of recurring cases. The maternal virus is probably reactivated during pregnancy, which results in recurrent infection.^4^ Fetal transmission is not totally prevented by the presence of maternal antibodies to cytomegalovirus, but these seem to have a protective effect against fetal disease.^9^ Congenital lesions are more serious when associated with primary infection .^3^ It has been reported that 10% of fetuses are symptomatic at birth, 20% to 30% do not survive due to severe lesions, and the great majority present delayed manifestations like bilateral neurosensory deafness and mental retardation, affecting 5% to 15% of asymptomatic neonates.^10^

The most frequently used methods for detecting immunoglobulin M and immunoglobulin G antibodies are the indirect immunofluorescence test and the enzymatic immunoassay (ELISA). The presence of a specific immunoglobulin M antibody suggests active infection.^11-13^ In 1985, Stagno and Whitley ^10^ performed a diagnostic study of cytomegalovirus in maternal blood using the ELISA and radio-immunoassay techniques to detect immunoglobulin M antibodies and reported approximately 95% specificity and 70% sensitivity.

The polymerase chain reaction is a molecular biology technique in which the production of large amounts of specific DNA fragments is induced from very low concentrations of complex substrates.^14^ The high sensitivity of the polymerase chain reaction allows the detection of very low amounts of viral particles (DNA or RNA).^15,16^ In 1992, Warren et al.^17^ reported on a study in which the polymerase chain reaction sensitivity was 89.2% and its specificity was 95.8%. The polymerase chain reaction is performed using a specific selection of *primers* (nucleotide sequences). Appropriate conditions provide results comparable to tissue culture inoculation, a method that is considered to be the gold standard for the diagnosis of cytomegalovirus infection .^18,19^

In 1995 and 1998, Revello et al.^20,21^ performed a study to select a test that would provide early and precise diagnosis of cytomegalovirus infection, using specific immunoglobulin M, polymerase chain reaction in amniotic liquid and viral culture. The purpose was to obtain a test that could reduce the time interval for the maternal and fetal infection diagnosis. Maternal diagnosis was based on seroconversion, clinical symptoms and increased liver enzymes. The following rates of sensitivity were reported: 69.2%, 76.9% and 55.5%, respectively. The authors concluded that a combination of highly sensitive techniques could improve prenatal diagnosis.

Studies that compare the performance of serological tests with the polymerase chain reaction test to diagnose cytomegalovirus infection could help in determining maternal serological validity. If infection is present it may allow the assessment of fetal risk of developing disease.

## OBJECTIVE

The purpose of this study was to assess the accuracy of the ELISA serological test in comparison with the polymerase chain reaction in maternal blood to diagnose cytomegalovirus infection.

## METHOD

The study sample consisted of 243 pregnant women with an indication for fetal blood sampling, who were being attended at the high-risk outpatient clinic (Pré-Natal Especializado, Universidade Estadual de Campinas). The minimum gestational age for collecting the blood sample was 21 weeks. The indications for fetal sampling were: diagnosis of fetal malformation via ultrasound; suspected congenital infection (cytomegalovirus, toxoplasmosis, rubella); antecedents and/or suspicion of chromosomal disease, fetal-maternal isoimmunization or intra-uterine growth restriction.

The polymerase chain reaction test results were used as the gold standard test^14^ and the ELISA serological test was the diagnostic test assessed . The gold standard defined a true clinical condition in a population.^22,23^ The control variables were: maternal age (years), race, marital status, number of years of education, number of sexual partners, number of gestations, fetal blood sampling indication and clinical symptoms. The participants in this study underwent cordocentesis. A peripheral venous blood sample of 10 ml was collected for diagnostic tests immediately before cordocentesis.

Two diagnosis tests were used in this study: 1. The serological ELISA test was performed using the ETI-Cytoc-M-Reverse commercial kit (Sorin Biomedical, Italy), read by the ETI-system reader (Sorin Biomedical, Italy). Automatic calculation was carried out using the ETI-system software; 2. The nested form of polymerase chain reaction was performed via direct visualization in 2% agar gel, following a method described by Saiki et al., 1985^14^, using appropriate equipment (DNA Thermal Cycler, Perkin Elmer/Cetus, Norwalk, Conn., USA).

Statistical analyses were done using Fish-er's exact test, via the SAS software, version 8.2 (SAS, 1999).^24^

## RESULTS

The majority of the women were between 21 and 30 years of age (51.4%). Their average age was 26 years. Most of them were white, had a single partner (married or living together), and were either at secondary school or had completed secondary school. During the interview, they reported one or two sexual partners : 50.6% and 30.5%, respectively. 70.4% reported at least two pregnancies and 29.6% had had just one. Ten of the patients (4.1%) reported symptoms that might have been related to cytomegalovirus.

The distribution of patients according to the indications for testing were: malformation of the central nervous system (25.5%), suspected toxoplasmosis (25.5%), Rh isoimmunization (14.8%), other malformations (12.8%), non-immune hydrops (3.3%), cytomegalovirus infection (0.8%), rubella (1.6%), intra-uterine growth restriction (1.6%), and other indications (12.8%). A positive serological immunoglobulin M test for cytomegalovirus in two patients indicated cytomegalovirus infection. The other indications were cardiac, thoracic and gastrointestinal tract malformations; maternal age; antecedents of fetal malformation or chromosome abnormality; and altered nuchal translucency.

The results of the diagnostic tests were: immunoglobulin G reagent 94.6%; immunoglobulin M reagent 0.8%; positive polymer-ase chain reaction 21.9%.

The prevalence of previous infections, as measured by positive immunoglobulin G, was 94.6% in the pregnant population of this study. As age increased, it was observed that there was an increase in the frequency of positive immunoglobulin G.

In the sample studied, there were two cases of positive immunoglobulin M (0.8%). One of these patients was 39 years old, non-white and married, with incomplete secondary education, one sexual partner, five previous pregnancies and reported symptoms that may have been related to cytomegalovirus infection. She had undergone serological examination at another hospital: specific immunoglobulin M and immunoglobulin G for cytomegalovirus. The other positive immunoglobulin M patient was 17 years old, white and single, with completed lower school level and one sexual partner. She was primigravida and had no clinical symptoms that could be associated with cytomegalovirus. She was referred because of a serological test with specific positive immunoglobulin M against toxoplasmosis.

When the polymerase chain reaction was used as a marker for active cytomegalovirus infection, 21.9% of the patients showed viral replication during pregnancy.

The polymerase chain reaction was positive for 6 of the 10 patients who reported clinical symptoms. This result was statistically significant. The two suspected cytomegalovirus cases presented positive polymerase chain reaction.

In the group of 49 women with positive polymerase chain reaction there were eight fetuses with positive polymerase chain reaction, two of which with documented maternal and fetal cytomegalovirus infection (fetal immunoglobulin G and immunoglobulin M positive). Among the six other cases, four had central nervous system malformation, one had positive immunoglobulin M to toxoplasmosis and there was one case of Rh isoimmunization. There was no child examined at birth with signs or symptoms associated with cytomegalovirus infection.

Table 1 relates the serological test results to the polymerase chain reaction results, in order to calculate sensitivity, specificity, positive predictive value, negative predictive value and likelihood ratio. Forty-six cases were found to have positive immunoglobulin G. The sensitivity of this diagnostic test was 94% when compared for the 49 positive polymer-ase chain reaction maternal blood samples. With regard to the 174 negative polymerase chain reaction cases, 10 cases were immunoglobulin G negative and the specificity was 6% (Table 1).

**Table 1:** Polymerase chain reaction test results for maternal blood samples, according to immunoglobulin G (IgG) test results

Maternal immuniglobulin G	Positive polymerase chain reaction	Negative polymerase chain reaction
Positive	46	164
Negative	3	10
**Total**	**49**	**174**

*Sensitivity = 46/49 = 93.88 % (95% confidence interval: 82-98%); Specificity = 10/174 = 5.75 % (95% confidence interval: 3-11%); Positive predictive value = 46/(164 + 46) = 21.90%; Negative predictive value = 10/(3 + 10) = 76.92%; Positive likelihood ratio = 1.00; Negative likelihood ratio = 1.07*

There were two cases of positive immunoglobulin M with positive polymerase chain reaction, and 47 cases of positive polymerase chain reaction but negative immunoglobulin M.

## DISCUSSION

The purpose of this study was to assess the accuracy of the serological test for diagnosing cytomegalovirus infection using the ELISA method in comparison with the polymerase chain reaction test. The results demonstrated that the serological tests had a low diagnostic performance in identifying cytomegalovirus infection in pregnant women. The fact that the serological tests showed a reduced diagnostic performance when compared to the polymerase chain reaction test is important because it means that a pregnant woman who is nonreactive to immunoglobulin M for cytomegalovirus may still be undergoing viral replication through recurrent infections or viral reactivation.

Approximately 5% of this population were susceptible to primary infection although they were immunoglobulin G negative. Cytomegalovirus serum prevalence increases as age increases and reaches its maximum level after the age of 25.^2^ In fact, in this study, most of those who had a previous history of cytomegalovirus infections were over 31 years old.

One important finding in this investigation was that six out of ten women that reported symptoms associated with cytomegalo-virus infection were polymerase chain reaction positive. Nonetheless, two women with positive immunoglobulin M denied having symptoms related to cytomegalovirus infection. We argue that if the real value of the polymerase chain reaction is demonstrated to be an identifier of active viral infection, it should then be requested upon clinical suspicion.

There are limitations to the interpretation of the test results for immunoglobulin M, and these should be kept in mind. The response of the specific immunoglobulin M antibody is not restricted to primary infections, as reactivation and reinfection may also cause an increase in immunoglobulin M titers.^13^ Other disadvantages include false negative results and false positive results due to low titers resulting from cross-reaction to rheumatoid factor.^25^

In this study, the polymerase chain reaction was used as the gold standard for infection diagnosis. This was because a positive polymerase chain reaction test signifies viral replication and detects pregnant woman at high risk of cytomegalovirus infection and transmission to the fetus.

Our attention was drawn to the fact that 15 of the 62 patients who were suspected of toxoplasmosis were polymerase chain reaction positive for cytomegalovirus. These women were referred because they presented a serologically positive immunoglobulin M test, detected by immunofluorescence against toxoplasmosis. We question whether this technique is sufficiently highly sensitive to detect small amounts of specific immunoglobulin M for toxoplasmosis. It might really represent an immune response to cytomegalovirus reactivation, since the polymerase chain reaction test was positive for one fourth of these patients. There is one reported case in which a woman in her 27^th^ week of pregnancy was diagnosed as having double seroconversion for cytomegalovirus and *Toxoplasma gondii*.^25^ This leads to the idea that when toxoplasmosis is suspected, the patient should also be tested for cytomegalovirus.

The ELISA test for specific immunoglobulin M, used for detecting viremia, demonstrated lower sensitivity in comparison with the polymerase chain reaction. The sensitivity was 4%, much lower than was found in the studies of Stagno and Whitley,^10^ Griffiths et al.^26^ and Donner et al.^27^ These authors obtained sensitivity levels that ranged from 20% to 80% at different gestational ages. They were, however, studying patients with a clinical suspicion of cytomegalovirus infection.

The serological tests using the immunoglobulin G reagent were helpful in determining cytomegalovirus seroprevalence and antecedents of previous infections. However, the specific immunoglobulin M showed very little relationship with viral replication regarding active and recurrent infections, since it was positive in only two of the 49 cases of positive polymerase chain reaction for cytomegalovirus. According to a study conducted by Stagno et al .,^22^ 73% of the pregnancies with primary infection and 11% of the pregnancies with secondary infections were diagnosed using immunoglobulin M on a group of patients with clinical suspicion of cytomegalovirus infection.

When comparing serology with the polymerase chain reaction for cytomegalovirus diagnosis, it is important to remember that serology is a diagnostic test that detects circulating antibodies and identifies the history of previous infections through immunoglobulin G and acute infections using immunoglobulin M. The polymerase chain reaction, on the other hand, is a diagnostic test that detects the presence of the DNA virus within the cell .^28^ A positive polymerase chain reaction result during pregnancy identifies patients who are undergoing viral replication within the cell but does not clarify the risk for disease development and fetal transmission.^29^

It is important to know whether viremia detected using a molecular technique signifies high risk of fetal transmission and, if fetal transmission takes place, how seriously the fetus would be damaged. Measurement of the viral load, the quantity of viral particles per milliliter in the analyzed material, would clarify the information obtained by a positive polymerase chain reaction result. Aitken et al.^29^ conducted a study that showed that viral loads found in patients with primary infections were higher than those in patients with recurrent infections. Since recurrent infections are more common during pregnancy, the viral load may be enough to detect the risk of vertical transmission.

Serology was ineffective in diagnosing pregnancies with a risk of infection and fetal transmission when the polymerase chain reaction was positive and the immunoglobulin G and immunoglobulin M were negative. Three of the 49 patients with positive polymerase chain reaction had negative immunoglobulin G, and immunoglobulin M was negative in these cases. In such cases, primary cytomegalovirus infection was detected through the polymerase chain reaction.

Therefore, pregnant women with a risk of cytomegalovirus fetal transmission could be identified using the polymerase chain reaction test for viral replication detection and, consequently, adequate follow-up could be established in order to monitor the fetus for infections and sequelae.

It is important to remember that the diagnostic evaluation tests were carried out using a single blood sample. Serology would have shown better levels of sensitivity if blood samples had been collected serially and the time intervals had been related to the appearance of immunoglobulin G and immunoglobulin M.

The results from this study showed that serological tests have a low capacity for diagnosing and tracking pregnancies with cytomegalovirus infection and a risk of fetal transmission, in comparison with the polymerase chain reaction. A correct diagnosis of the risk of fetal transmission after positive polymerase chain reaction could be complemented by serial serological analysis of the patient and/or study of antigenemia and viral load. In the future, techniques based on molecular biology will set the standards for detecting cytomegalovirus infection in pregnant women.

## CONCLUSION

The accuracy of the serological tests for the diagnosis of cytomegalovirus infection was lower than that of the polymerase chain reaction. The significance of the positive polymer-ase chain reaction results identified in this study remains to be completely understood.
